# Health-related quality of life 2 years after pedicle subtraction osteotomy for sagittal imbalance: a single-center experience of 65 patients

**DOI:** 10.1007/s00701-023-05787-0

**Published:** 2023-09-16

**Authors:** Pierre-Pascal Girod, Sara Lener, Nikolaus Kögl, Sebastian Hartmann, Anto Abramovic, Laura Krismer, Markus Santer, Martin Ortler, Claudius Thomé

**Affiliations:** 1 Department of Neurosurgery, Medical University of Innsbruck, Anichstrasse 35, 6020 Innsbruck, Austria; 2 Department of Neurosurgery, Klinik Landstraße, Vienna, Austria

**Keywords:** Health-related quality of life, Patient satisfaction, Pedicle subtraction osteotomy, PSO, Sagittal imbalance, Adult spinal deformity

## Abstract

**Purpose:**

Pedicle subtraction osteotomy (PSO) as an invasive procedure with high reoperation and complication rates in an often elderly population has often been questioned. The purpose of our study was to evaluate the impact of PSO for sagittal imbalance (SI) on patient-reported outcomes including self-reported satisfaction and health-related quality of life 2 years postoperatively.

**Methods:**

Consecutive patients who underwent correction of their spinal deformity by thoracolumbar PSO were assessed using self-reporting questionnaires 2 years postoperatively. Outcome was measured by visual analogue scale (VAS) for back and leg pain, Oswestry Disability Index (ODI), and EQ-5D scores. Additionally, a Patient Satisfaction Index (PSI) rated in four grades (A: very satisfied to D: not satisfied), walking range, and the Timed Up and Go (TUG) Test were evaluated.

**Results:**

Sixty-five patients were included, and each parameter was assessed preoperatively and 24 months postoperatively. The intervention led to significant improvements in back pain (8.1 ± 1.2 vs. 2.9 ± 1.9; *p* < 0.001), as well as ODI scores (57.7 ± 13.9 vs. 32.6 ± 18.9; *p* < 0.001), walking range (589 ± 1676 m vs. 3265 ± 3405 m; *p* < 0.001), and TUG (19.2 s vs. 9.7 s; *p* < 0.05). 90.7% of patients (*n* = 59/65) reported a PSI grade “A” or “B” 24 months postoperatively.

**Conclusion:**

Patient satisfaction 24 months after PSO for SI is high. Quality of life improved significantly by restoring sagittal balance.

## Background

Sagittal imbalance (SI) is commonly related to adult spinal deformity. Etiologies include primary deformity, such as scoliosis (degenerative and idiopathic), as well as secondary deformity, i.e., posttraumatic kyphosis and iatrogenic flat back syndrome. In the last decade, SI became a well-known cause of severe back pain and decreasing mobility, resulting in low health-related quality of life. Furthermore, patients commonly experience severe functional disability. As conservative treatment options fail in most patients, surgical interventions aiming to restore sagittal balance have increasingly gained importance [3]. Correction is typically accomplished by extensive instrumentation, frequently combined with osteotomy. While the surgical procedure has to be adapted to the amount of correction, pedicle subtraction osteotomy (PSO) is one of the most commonly performed interventions and results in sagittal correction of about 30° [13]. The PSO procedure was first described in 1985, aiming to correct kyphotic spinal deformities and was originally used in the ankylosed spine [25]. The principle is a closed wedge osteotomy with shortening of the dorsal structures, obtained by resection of the lamina, the facet joints, and the pedicles or the additional resection of the superior disc. Although the method is technically challenging, previous studies reported satisfactory clinical outcomes and improved quality of life by restoring sagittal alignment [2, 4, 13, 14, 16]. PSO and PSOplus (meaning an additional discectomy in the proximal adjacent level of the PSO) according to the classification of Schwab grades 3 and 4 [19] as invasive procedures with high reoperation and complication rates in an often elderly population have often been criticized and the benefit for the patients has been questioned [1, 6, 13]. Studies focusing on patient self-reported outcome after implementation of PSO for SI are lacking. Therefore, the purpose of this study was to investigate patient satisfaction and health-related quality of life (HRQOL) in patients suffering from SI and treated by a PSO.

## Material and methods

### Patient data

This study presents data from a prospective observational patient registry. Clinical management was not delayed or altered by participation in this study. The study was approved by the Local Ethics Committee (AN2014-0234) in accordance with the ethical principles originating from the Declaration of Helsinki and in compliance with Good Clinical Practice. Sixty-five consecutive patients, who underwent PSO or PSOplus in the thoracolumbosacral spine for SI between January 2011 and May 2018 at the authors’ institution, were enrolled. A full data set was obtained in all patients. Indication for surgery was determined in an interdisciplinary spinal case conference. The spino-pelvic parameters were assessed according to the FBI method of Le Huec et al. [13]. All patients were postoperatively observed and monitored for at least 24 h at the IMCU/ICU. Orthoses were not systematically prescribed. Demographic data as well as preoperative and postoperative clinical parameters were collected using the patient’s electronic chart. Furthermore, the occurrence of intraoperative and perioperative complications, as well as causes for revision surgery within 24 months postoperatively, was documented.

### Patient-reported outcome

Patient-reported outcome measures included visual analogue scale (VAS) for leg and back pain, Oswestry Disability Index (ODI), and EQ-5D-5L plus EQ-5D-VAS. The ODI is a widely used, simple, and quick condition-specific outcome measure in patients with spinal disorders. Zero to 20% indicate minimal disability, 20–40% moderate disability, 40–60% severe disability, 60–80% very serious disability, and 80–100% bed-bound disability [24]. The EQ-5D-5L is a simple re-runnable questionnaire, which is composed of five questions regarding mobility, self-care, usual activities, pain/discomfort, and anxiety/depression. The EQ-5D index, calculated from a specific algorithm, is subsequently subtracted from 1.000. The EQ-5D-VAS thermometer asks patients to mark their health status (0–100) on the day of the assessment [18]. Furthermore, walking range (in meters), Timed Up and Go Test (TUG in seconds [s]) [8], representing a performance-based functional test, objectively quantifying the physical ability of walking, and the NASS Patient Satisfaction Index (PSI) rated in four grades (A: very satisfied to D: not satisfied) were assessed [7]. Scores were obtained preoperatively and 3 months, 12 months, and 24 months postoperatively. All clinical outcome measures were determined during routine outpatient visits by the patients with assistance of a study nurse if necessary.

### Surgical procedure

Patients were placed in a prone position after induction of general anesthesia with the goal to place the vertebral body to be osteotomized directly at the pivot point of the operating table [10]. Standard posterior exposure of the spine was realized and bony structures were fully visible. Pedicle screws were placed in a standardized manner, at least two levels above and two levels below the aimed PSO level. If necessary, Smith-Petersen Osteotomy (SPO) [23] and transforaminal lumbar interbody fusion (TLIF) were performed at additional levels. A laminectomy with cranio-caudal undercutting at the index level was performed according to the classification of Schwab et al. [19]. Additionally, the transverse process of the osteotomized level and the facet joints of the remaining adjacent and instrumented segments were resected. Pedicles were then opened by using multiple specific dilators. Cancellous bone was progressively pushed anteriorly to create a solid anterior fusion mass. Pedicles were resected with differently shaped osteotomes (PSO Osteotomy Set, Medtronic, Dublin, Ireland). If necessary, the adjacent superior disc was removed (PSOplus). The posterior vertebral wall was resected in two steps with a special posterior wall impactor. The osteotomy was then closed in a gentle manner by adjustment of the OR table hinge remote controlled and under continuous visualization of the dural sac and the descending nerve roots [10]. One or two satellite rods were usually inserted to span the osteotomy [21].

### Statistical analysis

All patients with complete initial data were considered for the retrospective analysis. Continuous variables are expressed by mean ± standard deviation (SD). The Kolmogorov–Smirnov test was used for testing normal distribution. The unpaired Student *t*-test and Mann–Whitney *U* test were performed to analyze differences in clinical and demographic characteristics and in clinical outcome variables. Frequencies were compared by the chi-square and Fisher’s exact tests. A *p*-value < 0.05 was considered as statistically significant. All statistical evaluations were performed with SPSS Version 21.0 (IBM Corp. Released 2012. IBM SPSS Statistics for Mac OS X, Version 21.0, NY: IBM Corp.). Figures and tables were designed using Microsoft Excel (Version 15.36 for Mac OS X, Microsoft Corporation 2017, Redmond, USA).

## Results

### Clinical details

The data of sixty-five prospectively included patients matching the inclusion criteria and completing their 24-month follow-up were analyzed. Demographic details and patient characteristics are outlined in Table 1. Thirteen patients (20%) experienced intraoperative complications, with 11/13 cases (84.6%) of incidental durotomy and 2/13 cases (15.4%) of extensive blood loss (> 5000 mL). Eleven patients (16.9%) demonstrated perioperative complications: 2/11 (3.1% overall) developed a superficial wound infection, 4/11 (6.2% overall) experienced a postoperative thrombosis, and 5/11 (7.7%) demonstrated postoperative neurological worsening. Within 24 months, 4/65 patients (6.2%) were treated for proximal junction kyphosis (PJK), whereas 6/65 patients (9.2%) received revision surgery due to hardware failure.

**Table 1:** Demographic details (*ASA*, American Society of Anesthesiologists; *n*, population; *PSO*, pedicle subtraction osteotomy; *SD*, standard deviation)

Sex, *n* (%)	Female	40 (62)
	Male	25 (38)
Age	Years (SD)	66.2 (± 10.9)
ASA, *n (%)*	°1	4 (6)
	°2	26 (40)
	°3	34 (52)
	°4	1 (2)
Prior surgery, *n (%)*	Yes	56 (86)
	No	9 (14)
Level of PSO, *n (%)*	Thoracal	12 (19)
	Lumbar	53 (81)
Operative method, *n (%)*	PSO	38 (59)
	PSOplus	27 (41)

### Self-reported, health-related quality of life

**Fig. 1:**
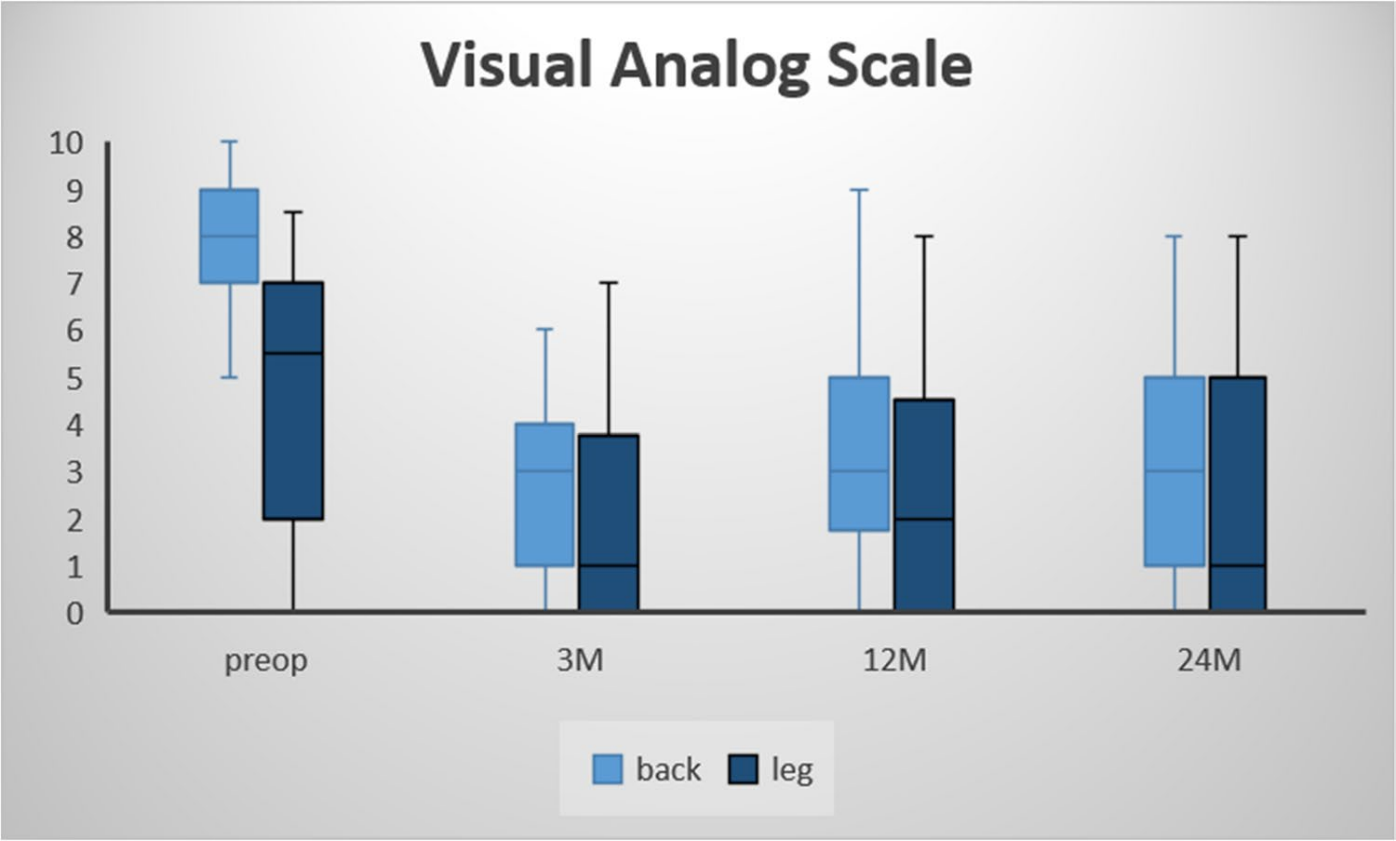
VAS scores for back and leg pain over time, preoperatively to 24 months postoperatively

**Fig. 2:**
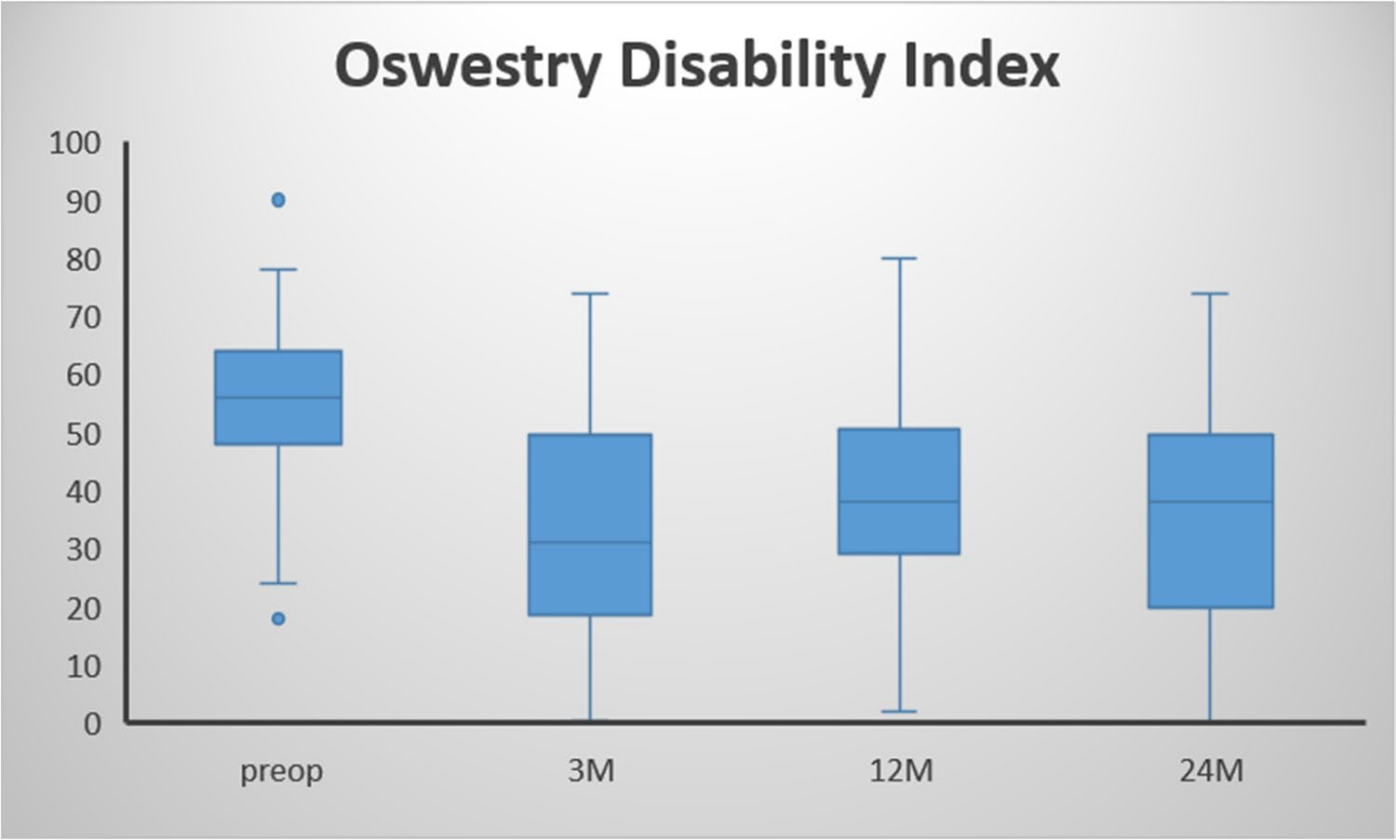
ODI values over time, preoperatively to 24 months postoperatively

**Fig. 3:**
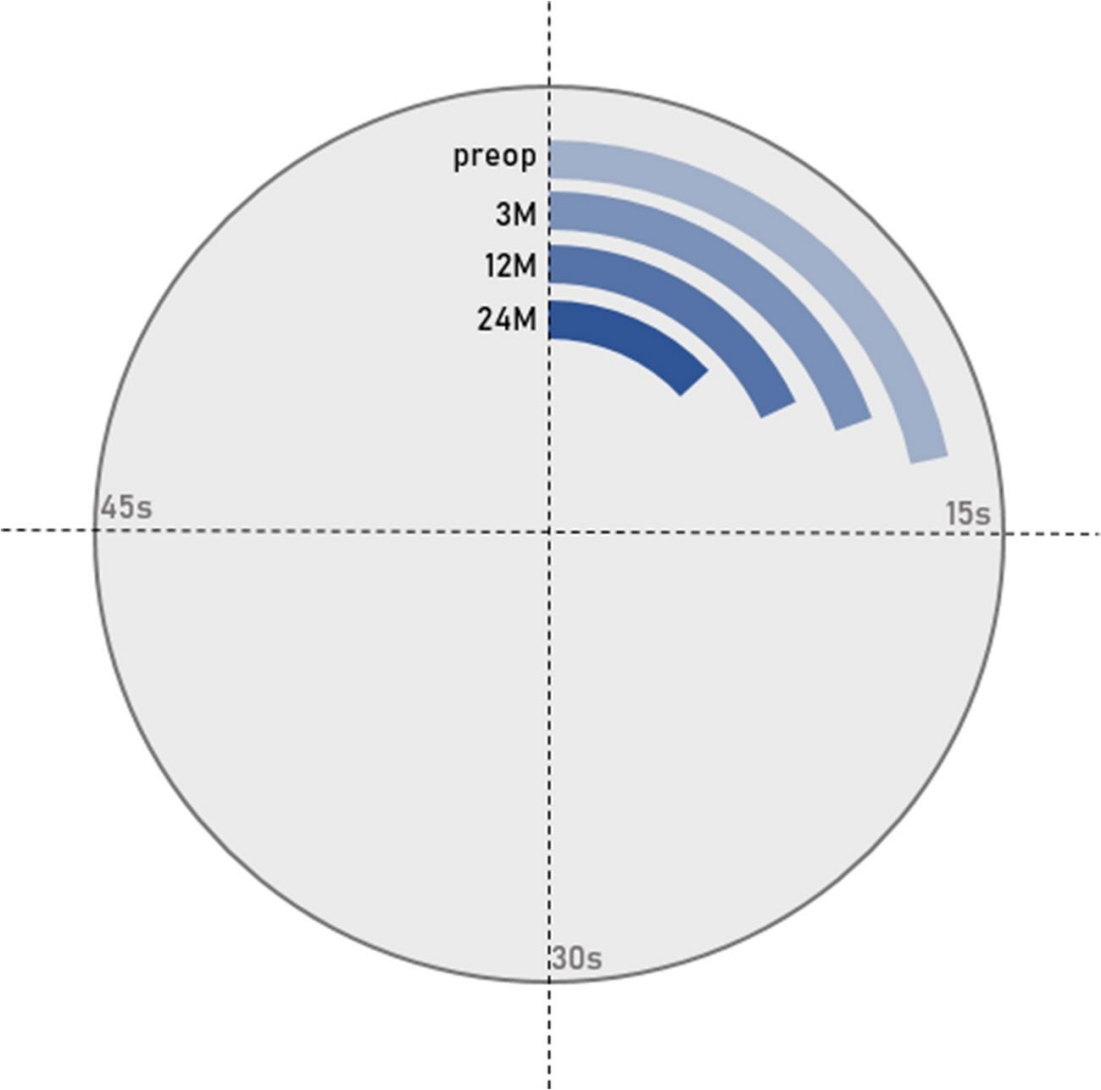
Changes in EQ-5D-5L (values × 100) and EQ-5D-VAS (0–100%) preoperatively to 24 months postoperatively

**Fig. 4:**
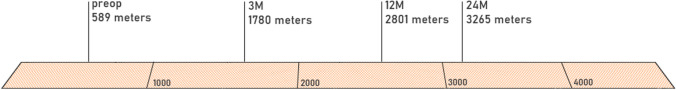
Walking range and TUG test at four different time points between preoperative and 24-month postoperative evaluation

**Fig. 5:**
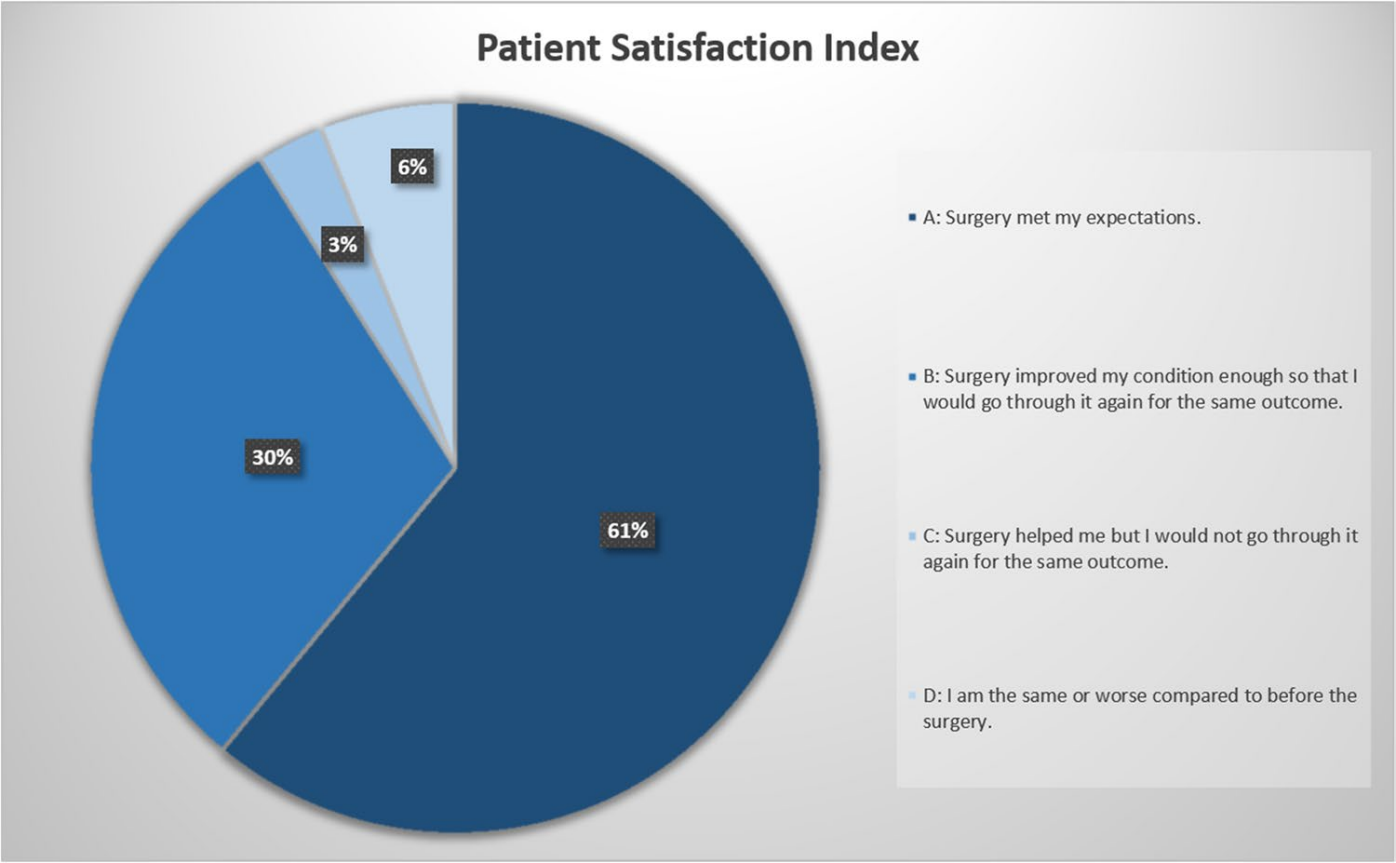
Distribution of the PSI 24 months postoperatively. A: Surgery met my expectations; B: Surgery improved my condition enough so that I would go through it again with the same outcome; C: surgery helped me but I would not go through it again for the same outcome; D: I am the same or worse compared to before surgery

The VAS for back and leg pain demonstrated significantly lower values comparing pre- to 24-month postoperative data (81 ± 12 vs. 29 ± 19; *p* < 0.001; 46 ± 29 vs. 20 ± 22; *p* < 0.001; respectively; Fig. 1). Surgery also significantly improved ODI scores (57.7 ± 13.9 vs. 32.6 ± 18.9; *p* < 0.001; Fig. 2) and EQ-5D values (0.48 ± 0.21 vs. 0.67 ± 0.26; *p* = 0.028; Fig. 3). The self-reported health state, as a part of the EQ-5D evaluation, demonstrated lower values during the postoperative course, but no statistically significant differences (50.6 ± 21.5 vs. 61.6 ± 15.8; *p* = 0.109). The patient-reported walking range and the TUG test improved significantly in the period between the preoperative and 24-month postoperative evaluation (589 ± 1676 m vs. 3265 ± 3405 m; *p* < 0.001 and 19.2 s vs. 9.7 s; *p* = 0.017; respectively; Fig. 4). 90.7% of patients (*n* = 59/65) reported a PSI grade “A” or “B” 24 months postoperatively, i.e., they would have the operation performed once again with the same outcome (Fig. 5). A correlating trend of worse preoperative and improved postoperative scores could be shown (*p* = 0.161). There was no correlation of sex or gender and HRQOL outcome.

## Discussion

The prospective evaluation of health-related quality of life 24 months after PSO for SI demonstrated excellent improvement of quality of life, pain, and functionality. Patient satisfaction was very high, as 90.7% of patients were satisfied with their outcome 2 years postoperatively.

A direct correlation between low HRQOL and SI has already been described [15]. Causes for this and main indications for deformity correction surgery include significant functional impairment and intractable pain. Spinal osteotomies seem to be effective methods in restoring sagittal balance and improving HQOL in patients with severe thoraco-lumbar deformities [2, 13, 14]. Their use, however, has often been criticized due to a relatively high complication rate in an elderly population. Nevertheless, there is a lack of studies reporting on the patient self-reported HRQOL and on patient satisfaction following thoracolumbar PSO for SI.

### Complications

Numbers presented in this study are comparable to recently published data, where intraoperative and perioperative complications are reported in up to 55%, mostly independent from the surgeon’s experience [1, 6, 20]. This may be explained by the fact that most patients receiving treatment for SI are over 50 years of age and commonly had prior surgery [6]. Perioperative complication rates and causes, as well as the incidence of hardware failure and PJK, coincide with recent reports [5, 22]. According to these findings, the indication for such an invasive operation should be comprehensively evaluated and the patients should be well informed before surgery. This is particularly applicable in patient cohorts like the treated group, characterized by advanced age and a significantly higher risk due to often multiple previous operations.

### Improvement of pain

We could demonstrate a significant improvement of pain, and the minimal clinical important difference (MCID) of at least 28 points was reached immediately [17]. This coincides with previous reports, where realignment surgery has been shown to improve radicular symptoms as well as back pain [22].

### Improvement of disability

Significant improvement could also be demonstrated for ODI, as results improved even more than the reported MCID of 10 points [17]. As a further instrument for quantification, the TUG test showed a significant improvement during the postoperative course. The TUG test represents a performance-based functional test that objectively documents and quantifies the physical ability of walking and is reported to be sensitive and appropriate for assessing postoperative changes after instrumented fusion surgery. The MCID has been reported at 3.4 s [9, 12]. Our results correspond with previous studies, demonstrating that even mild SI may be detrimental for the patient’s functional status, and the correction of the sagittal alignment shows an early and significant improvement [11, 22]. Furthermore, our data may be interpreted to show even better benefit of the surgical procedure, the higher the preoperative disability was rated.

### Improvement of health status

EQ-5D-5L values, accounting for the generic health status, showed significant improvement over time, reaching significantly more than the MCID of 0.03 points at 24 months (0.48 ± 0.21 vs. 0.67 ± 0.26) [24]. Similar results could be shown for the EQ-5D-VAS, where patients are asked to rate their subjective health status and clearly reached the previously reported MCID of 10.5 points [24]. Moreover, the individual walking range reported by patients significantly improved over time, showing a nearly sevenfold increase between the preoperative and 24-month postoperative evaluation.

### Limitations

Limitations of the study include the small patient cohort, the single-center management and bias, and the short period of follow-up. Nevertheless, our data might well be conclusive, as the majority of complications and re-operations — and the resulting disability — is known to occur within the first 2 years after correction surgery [21].

As the population ages, the rate of surgeries performed for adult spinal deformity will continue to rise. Better understanding of sagittal deformities, technical improvements, and improved preoperative and postoperative care allow these surgeries to become highly complex. This, however, might be associated with a concomitantly increased risk of complications. Despite a reoperation rate of 15.4% and a complication rate of 20% intraoperatively and 16.9% postoperatively, however, more than 90% of patients were satisfied with the procedure.

## Conclusion

Despite the complex surgical procedure and a relatively high overall complication rate with PSO to restore sagittal balance, patient satisfaction is high, and quality of life improved significantly 24 months after surgery.
